# Scutellarin Alleviates Neuronal Apoptosis After Ischemia and Hypoxia via the HIF‐1α–CX3CR1 Axis

**DOI:** 10.1002/cns.71098

**Published:** 2026-08-19

**Authors:** Jingchun Pei, Bin Hu, Zhonghui Wen, Cheng Wan, Fuqing Zhang, Shaoxiang Li, Lei Wang, Zhigao Li, Zhiwei Tang

**Affiliations:** ^1^ Department of Neurosurgery The First Affiliated Hospital of Kunming Medical University Kunming China; ^2^ Department of Interventional Radiology The First Affiliated Hospital of Kunming Medical University Kunming China; ^3^ Department of Neurosurgery Dali Prefecture People's Hospital Dali China; ^4^ Department of Neurosurgery Dali People's Hospital Dali China

**Keywords:** apoptosis, CX3CR1, ischemic stroke, neuron, scutellarin

## Abstract

**Background:**

Stroke is a significant health threat characterized by high incidence, mortality, disability, recurrence, and complications. Over 70% of strokes are ischemic in nature. Reducing neuronal apoptosis following ischemic–hypoxic injury is crucial for effective treatment. This study investigates the direct neuroprotective effects of scutellarin after ischemia–hypoxia, clarifies the relationship between upregulated CX3CR1 expression and neuronal apoptosis, and elucidates the mechanism by which scutellarin regulates apoptosis through the HIF‐1α–CX3CR1 axis. This research provides a robust theoretical foundation for the clinical application of scutellarin in the management of ischemic stroke.

**Methods:**

A mouse model of transient middle cerebral artery occlusion (tMCAO) was established in vivo, and an oxygen–glucose deprivation/reoxygenation (OGD/R) model was constructed by culturing primary neuronal cells in vitro. The direct protective effects of scutellarin on neurons following ischemia and hypoxia were observed, along with its impact on neurological function and cerebral infarct volume. Changes in the expression of neuronal CX3CR1 and its effects on apoptosis after ischemia and hypoxia were determined using immunofluorescence, Western blotting, and CX3CR1 transgenic mice. Finally, quantitative PCR (Q‐PCR), immunofluorescence, and Western blotting were employed to investigate whether scutellarin modulates NF‐κB via the HIF‐1α–CX3CR1 axis and the JAK1–STAT1 pathway, thereby influencing neuronal apoptosis.

**Results:**

In vitro and in vivo experiments have demonstrated that scutellarin reduces neuronal apoptosis, thereby decreasing the infarct area and alleviating neurological deficits in MCAO mouse models. Following ischemia and hypoxia, the expression of CX3CR1 in neurons is upregulated, which mediates neuronal apoptosis. Scutellarin downregulates the expression of CX3CR1 by modulating HIF‐1α. Additionally, CX3CR1 can regulate neuronal apoptosis after ischemia through the NF‐κB/P65 and JAK1‐STAT1 signaling pathways.

**Conclusions:**

Our experimental results confirm that scutellarin regulates neuronal apoptosis following ischemia and hypoxia through the HIF‐1α‐CX3CR1 axis. These findings provide additional theoretical evidence for the clinical application of scutellarin and suggest potential therapeutic targets for the treatment of ischemic stroke.

## Graphical abstract

In this study, we established a mouse model of middle cerebral artery occlusion and cultured primary neurons to evaluate the protective effects of scutellarin against ischemia and hypoxia, specifically analyzing CX3CR1 expression and its role in apoptosis. The results demonstrated that scutellarin attenuates neuronal apoptosis via the HIF‐1α–CX3CR1 axis by modulating the NF‐κB and JAK1‐STAT1 pathways. These findings provide a robust theoretical basis for the clinical application of scutellarin and highlight potential targets for the treatment of ischemic stroke.

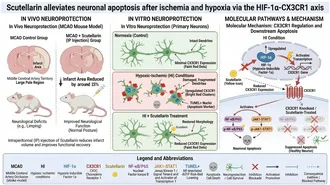

## 1 Introduction

The global increase in population aging has made stroke the second leading cause of mortality and the third leading cause of disability worldwide, with projections indicating it may soon claim the top spot. Stroke is primarily categorized as ischemic and hemorrhagic types, with epidemiological data indicating that ischemic stroke affects over 70% of stroke patients [1, 2]. Despite advances in ischemic stroke understanding, management is challenging. rt‐PA is the sole drug option. Prompt reperfusion is vital but risks reperfusion injury and hemorrhage [3, 4]. The development of neuroprotective strategies to protect brain cells from damage and extend the therapeutic window for thrombolytic therapy is a critical objective. The search for effective neuroprotective agents is an urgent clinical challenge.

The neuroprotective potential of natural compounds, especially scutellarin, has gained research interest due to its diverse biological activities in treating neurological and circulatory diseases [5, 6]. The protective effect of scutellarin on ischemic stroke primarily involves regulating microglial polarization, indirectly exerting neuroprotective effects by reducing the release of pro‐inflammatory cytokines [7]. Some studies have also found that scutellarin exerts a direct neuroprotective effect by reducing the expression or activity of iNOS [8]. The mechanism underlying the direct neuroprotective effect of scutellarin on neurons following ischemic stroke remains poorly understood. Further investigation into the mechanisms underlying the direct neuroprotective effects of scutellarin on neurons after ischemia is expected to provide stronger evidence for ischemic stroke treatment and holds significant clinical research value.

CX3CR1, a member of the G protein‐coupled receptor family, is critically involved in cellular regulation following central nervous system (CNS) injury, particularly in the regulation of neuronal apoptosis [9, 10]. Previous studies have shown that CX3CR1 is primarily expressed on microglia and plays a key role in regulating neuroinflammation and cell apoptosis. CX3CR1 also modulates cytokine release and intercellular signal transduction and further influences neuronal apoptosis by affecting the interactions between microglia and neurons [11]. In addition, the CX3CR1 signaling pathway exerts bidirectional regulatory effects in different neurodegenerative disease models, indicating the complexity of its regulatory role in neuronal apoptosis [12]. However, there is no relevant research on whether scutellarin can affect neuronal apoptosis by regulating the expression of CX3CR1 in neurons. Therefore, we used the middle cerebral artery occlusion (MCAO) model in mice, the oxygen–glucose deprivation/reperfusion (OGD/R) model of neurons, and the CX3CR1 transgenic mouse model to explore the direct protective effect of scutellarin on neurons and its mechanism of action. The results showed that the expression of CX3CR1 in neurons was upregulated after ischemia and hypoxia, which participated in the apoptosis process; scutellarin directly inhibited neuronal apoptosis by regulating the NF‐κB and JAK1–STAT1 signaling pathways through the HIF‐1α–CX3CR1 axis. This study not only provides a theoretical basis for the treatment of ischemic stroke with scutellarin but also suggests potential intervention targets.

## 2 Materials/Subjects and Methods

### 2.1 Laboratory Animals

In this study, healthy adult male and female SPF‐grade C57BL/6 mice and CX3CR1−/− mice (Product no. KOCMP‐13051‐Cx3cr1‐B6J‐VA) weighing 18–22 g and aged 8 weeks were utilized. These mice were sourced from Suzhou Cyagen Biosciences Inc. and were maintained on SPF feed from Beijing Keao Xieli Feed Co. Ltd. They were housed in a controlled environment at Kunming Medical University, with ad libitum access to food and water, under conditions of (20°C ± 2°C) and a 12‐h light–dark cycle. Animal experiments performed in the present study were approved by Kunming Medical University (Kmmu202220920) and were performed in accordance with international guidelines for animal studies and the guidelines of the Committee on Animal.

### 2.2 Establishment of the Middle Cerebral Artery Occlusion (MCAO) Model in Mice and Drug Treatment

Prior to the surgical procedure, mice were subjected to fasting and dehydration. Subsequently, they were anesthetized via intraperitoneal administration of0.1% sodium pentobarbital at a dosage of0.1 mL/10 g. Following weighing, the right common carotid artery (CCA) and the right external carotid artery (ECA) were individually exposed under microscopic visualization. A specialized suture plug designed for the mouse model of middle cerebral artery occlusion (MCAO) was carefully inserted into the internal carotid artery. The advancement of the suture was conducted gradually until encountering slight resistance, at which point it was securely ligated and immobilized to prevent dislodgment. After a 90‐min interval, the suture plug was gently retracted, and the mice's responses were meticulously monitored. Postoperatively, measures were taken to maintain the mice's body temperature. Upon complete recovery from anesthesia, the mice were placed back into their cages, each housed separately. Subsequently, under blind conditions, two evaluators assessed the mice at 24, 48, and 72 h post‐model establishment (Table S1), followed by the computation of the average score from both observers.

### 2.3 Purification and Identification of Primary Neuron Culture

Newborn fetal mice were aseptically prepared and decapitated, followed by the meticulous dissection of the pia mater under a dissecting microscope. Subsequently, the brain tissue was finely minced, enzymatically digested, and filtered through a 70‐μm cell sieve. The resulting cell suspension underwent two rounds of centrifugation, followed by resuspension, and the cell density was determined using an inverted microscope. The cell suspension, at a concentration of approximately 1 × 10^6^ cells/mL, was then seeded into a 6‐well plate for culturing. Following an incubation period of 2–4 h at 37°C with 5% CO_2_, the culture medium was completely exchanged with a neuron‐specific medium.

Primary neurons were cultured for 48 h before introducing Ara‐C into the culture medium. Following a 24‐h treatment, half of the medium was refreshed, with subsequent half‐medium changes every 3 days. After 9 days, the neuronal culture medium was discarded, and neurons were subjected to PBS washing, fixation, and blocking. Subsequently, anti‐MAP2 antibody was applied and left to incubate overnight at 4°C. Following PBS washing, a fluorescent secondary antibody was applied and left to incubate at room temperature in the dark. DAPI staining was then performed at room temperature in the dark. Fluorescence microscopy was utilized for observation and imaging, with subsequent quantification of MAP2‐positive and DAPI‐positive cells. Neuronal purity was determined using the formula: Neuronal purity = (MAP2(+)/DAPI(+)) × 100% (Figure S1).

### 2.4 PC‐12 Cell Culture

PC‐12 cells obtained from ATCC (Shanghai, China) were cultured in DMEM medium (Gibco) supplemented with 10% fetal bovine serum (Gibco) at 37°C in a 5% CO_2_ incubator. Upon reaching 80% confluence, the cells were detached using trypsin, reseeded into 6‐well plates, and allowed to adhere for 12 h. Subsequently, the cells, confirmed to be in optimal condition, were utilized for further experimentation.

### 2.5 Oxygen–Glucose Deprivation and Drug Treatment

Primary neuronal cells were subjected to OGD/R to mimic ischemic and hypoxic conditions. The cells were divided into three groups [1]: Normal control group, where cells received complete medium [2]; Model group, where cells were exposed to anoxia by replacing the medium with sugar‐free solution after PBS washes; and [3] Scutellarin treatment group, where cells were pre‐treated with 80 μM SCU for 4 h before anoxia. Following the establishment of the OGD/R model, cells in this group were cultured in complete medium supplemented with 80 μM SCU for 24 h. The duration of anoxia was 30 min for primary neurons and 4 h for the PC‐12 cell line.

### 2.6 Western Blotting

Following removal of the culture medium, rinse the cells in a six‐well plate before adding 100–200 μL of RIPA lysis buffer with 1% protease inhibitor per well, or 600 μL per culture flask. For mouse brain tissue, utilize 150–250 μL of lysis buffer per 20 mg of brain tissue. Subsequently, lyse the samples thoroughly on ice for 30 min, and then transfer the cell suspension and proteins into 1.5 mL EP tubes. Centrifuge at 15,000 rpm at 4°C for 15 min, retaining the supernatant post‐centrifugation. Determine the protein concentration using BCA (Pierce, Rockford, USA) following the manufacturer's protocol. Primary antibodies such as those against CX3CR1, HIF‐1α, NF‐κB, JAK–STAT, and β‐ACTIN were employed for protein detection and quantification.

### 2.7 Immunofluorescence

Cells were washed, fixed, permeabilized for 10 min, underwent antigen retrieval, and were subsequently blocked with 5% bovine serum. Mouse brain tissues were paraffin‐embedded and coronally sectioned. Following baking, dewaxing, and rehydration, the tissues underwent antigen retrieval, permeabilization, and blocking before being exposed to a mixed primary antibody for immunofluorescence. Incubation was conducted overnight at 4°C. Subsequent to washing, a fluorescent secondary antibody was applied and incubated at room temperature in the dark, followed by dark washes. Mounting was performed with an anti‐fluorescence quencher containing DAPI. Subsequently, observations and photomicrographs were captured using a fluorescence microscope, and image analysis was carried out using ImageJ software.

### 2.8 Flow Cytometry Analysis

Prepare a cell suspension at a concentration of 1 × 10^6^ cells per milliliter. Transfer 100 μL of the suspension into a 5‐ml flow cytometry tube and supplement it with 5 μL of Annexin V‐FITC, gently mixing the contents. Incubate the tube in darkness at room temperature (20°C–25°C) for 10 min. Just before loading onto the flow cytometer, introduce 10 μL of propidium iodide solution and gently mix. After a further 5 min, add 400 μL of PBS to the tube to resuspend the cells, and store it in darkness until analysis.

Flow cytometry analysis was conducted using fluorescence‐activated cell sorting (FACS). Annexin V‐FITC and propidium iodide (PI) were utilized as fluorophores emitting green and red fluorescence, respectively. Data analysis was performed with FlowJo version 10.9.

### 2.9 Cell Sequencing and Analysis

RNA was extracted from 10 pairs of neuronal cell samples, with transcriptome sequencing conducted on 7 pairs and whole transcriptome resequencing on the remaining 3 pairs. The RNA extraction utilized TRIzol reagent (Invitrogen, Carlsbad, CA, USA) following the manufacturer's protocol. The Limma package (version 3.48.3) was utilized for differential gene expression analysis between the model and control groups, as well as between the therapy and model groups, using a threshold of |logFC| ≥ 1 and *p* < 0.05, resulting in the identification of DEGs1 and DEGs2, respectively. The up‐regulated DEGs1 were compared with the down‐regulated DEGs2, and vice versa, to identify common DEGs. Enrichment analysis was then performed to identify functions and pathways related to cell death, with the genes involved designated as cell death‐related genes (CDRGs). Subsequently, enrichment analysis and expression patterns among different groups were investigated for the CDRGs.

### 2.10 Real‐Time Quantitative PCR

RNA was isolated from mice following Takara's instructions. Subsequently, reverse transcription and amplification were conducted using the TB Green qPCR method, adhering to the manufacturer's protocol (Table S2).

### 2.11 Nissl Staining

Following paraffin embedding of mouse brain tissue, coronal sections were prepared and subsequently dewaxed and hydrated. The sections were stained with methyl violet, washed, and then subjected to Nissl differentiation solution for 5 s to remove most of the staining under microscopic observation. Following dehydration, clearing, and mounting, the paraffin sections were examined and photographed using a light microscope.

### 2.12 Statistical Analysis

Statistical analysis and graphical representation were performed using GraphPad Prism 9.5. One‐way analysis of variance (ANOVA) followed by Tukey's multiple comparison test was utilized for data analysis. Data conforming to a normal distribution were assessed using *t*‐tests, while non‐normally distributed data were analyzed using the Mann–Whitney U test. Statistical significance was defined as a *p* value < 0.05. The ‘*n*’ values in the figure legends indicate the number of repetitions in both in vitro and in vivo experiments or the number of animals involved.

## 3 Results

### 3.1 Direct Protective Effect of Scutellarin on Neurons After Ischemia and Hypoxia

**FIGURE 1:**
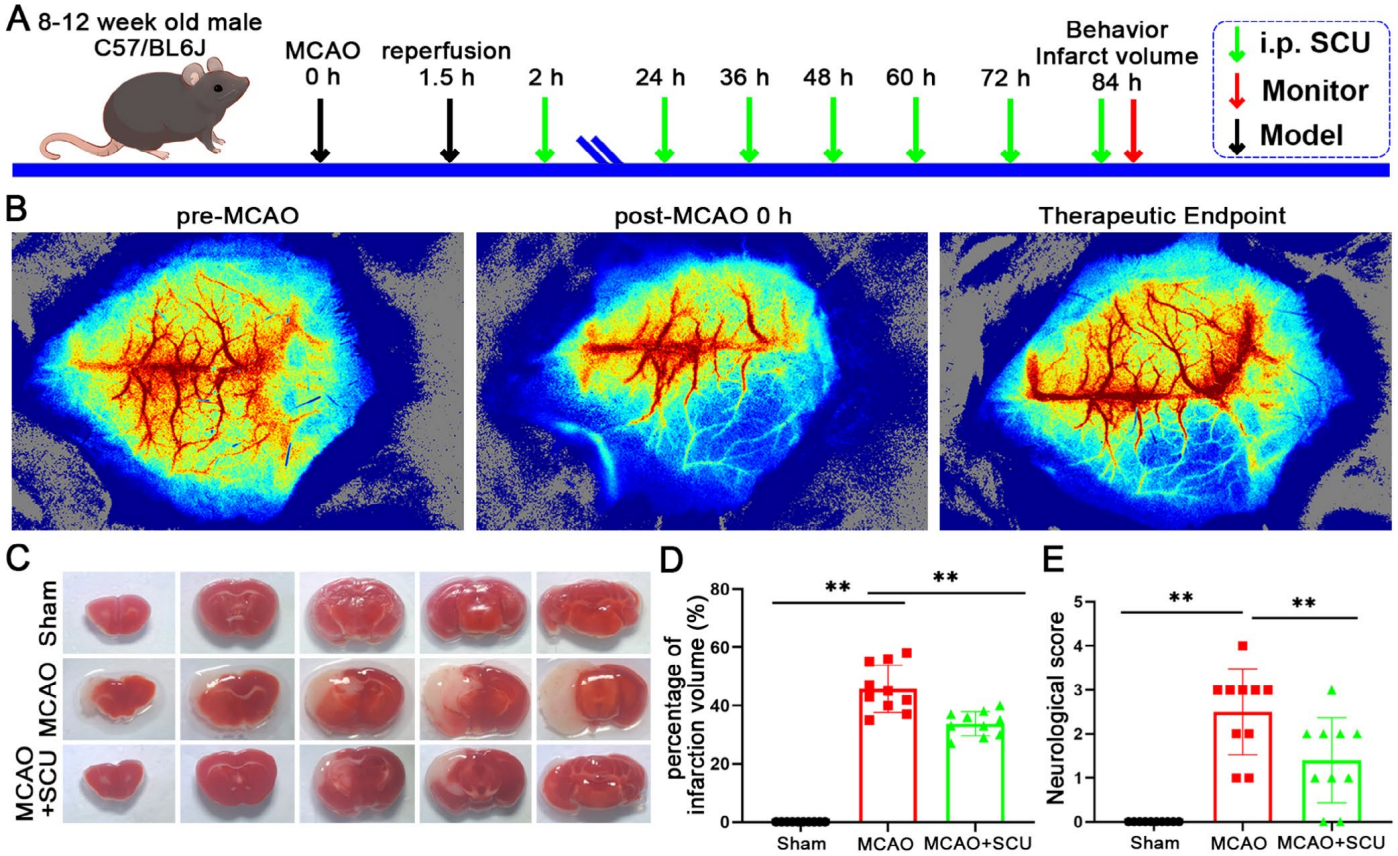
Direct protective effect of scutellarin on neurons after ischemia and hypoxia. (A) Experimental timeline outlining treatment protocols for scutellarin and TTC; neurological function score in tMCAO rats. (B) Representative laser speckle patterns in each group of mice. (C,D) The infarct area of the WT mouse brain 3 days after tMCAO. Represented by TTC staining, the infarcted area appears white. (E) The Longa score showed that treatment with scutellarin can significantly improve neurological function damage caused by cerebral ischemia–reperfusion (**p < 0.01, *n* = 10).

**FIGURE 2:**
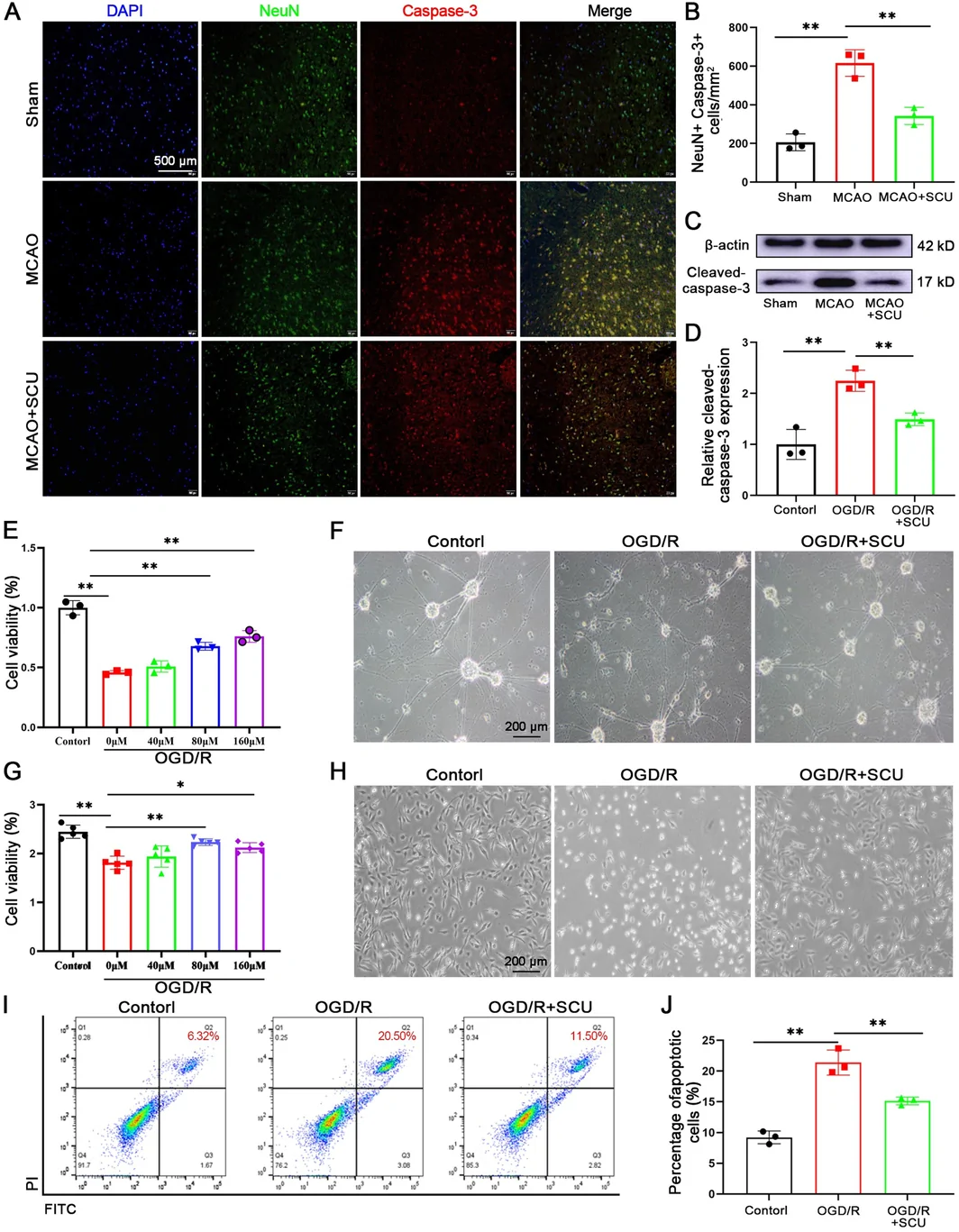
Direct protective effect of scutellarin on neurons after ischemia and hypoxia. (A,B) Fluorescence staining of NeuN/caspase‐3 showed that MCAO could lead to increased neuronal apoptosis in mice, and treatment with scutellarin (100 mg/kg) could reduce neuronal apoptosis caused by cerebral ischemia. (**p < 0.01, *n* = 3). (C,D) Western blotting showed that OGD/R can upregulate the expression of Cleaved caspase‐3, while treatment with scutellarin resulted in a decrease in Cleaved caspase‐3 expression. (E,F) Scutellarin treatment can improve the morphological changes of primary neuronal cells caused by OGD/R and increase cell viability. (G,H) Scutellarin can improve the morphological changes of PC‐12 cells caused by OGD/R and increase cell viability. (*p < 0.05、**p < 0.01, *n* = 3). (I,J) Flow cytometry analysis showed that OGD/R can increase apoptosis in PC‐12 cells, while treatment with scutellarin can reduce cell apoptosis (*p < 0.05, **p < 0.01, *n* = 3).

In the construction of the model in WT mice, three groups were established: Sham, MCAO, and SCU. The SCU group received intraperitoneal injections of scutellarin at a dosage of 100 mg/kg once fully awake post‐model induction, followed by twice‐daily injections for three consecutive days, based on previous experimental parameters. Cerebral blood flow (CBF) changes in mice post‐MCAO were assessed using laser speckle imaging before model induction, post‐suture removal, and pre‐sacrifice in the cerebellum (Figure 1A,B). Analysis of TTC‐stained sections revealed a significantly smaller infarct volume in the SCU (100 mg/kg) treatment group (32.97% ± 2.2%) compared to the untreated MCAO group (46.22% ± 5.07%), with a notable statistical difference (*p* < 0.01) (Figure 1C,D). Neurological function impairment in mice was evaluated using the Longa score (Figure 1F), showing a statistically significant difference (*p* < 0.01). Additionally, NeuN/caspase‐3 immunofluorescence double‐staining demonstrated an increase in NeuN and caspase‐3 double‐positive cells in the MCAO group (206 ± 44/mm2) compared to the control group, which decreased significantly post‐intraperitoneal scutellarin injection (343 ± 45/mm2), indicating a notable difference from the MCAO group (Figure 2A,B). These findings suggest that intraperitoneal scutellarin administration can mitigate cerebral infarction, ameliorate neurological function impairment, and reduce neuronal apoptosis induced by cerebral ischemia–reperfusion.

In vitro experiments were conducted by culturing primary neurons to establish three groups: The control group, the OGD/R group, and the SCU group. Observation under an inverted microscope revealed notable differences among the groups. The OGD/R group exhibited cellular aggregation, cell body swelling, reduced cell translucency, shrunken nuclei, irregular cell shapes, decreased intercellular connections, and disintegrated or bead‐like cell processes compared to the control group. Treatment with scutellarin mitigated these changes (Figure 2E,F). Similar observations were made in PC‐12 cells compared to primary neurons (Figure 2G,H). Protein analysis demonstrated a significant reduction in Cleaved‐caspase‐3 expression following scutellarin treatment, with a statistically significant difference compared to the OGD/R group (Figure 2C,D). Additionally, Annexin V‐FITC/PI double‐staining flow cytometry results indicated a marked increase in apoptotic cells in the OGD/R group compared to the control group. Treatment with scutellarin significantly decreased the proportion of apoptotic cells compared to the OGD/R group (Figure 2I,J). These findings collectively suggest that scutellarin treatment directly reduces neuronal apoptosis induced by OGD/R.

### 3.2 Neural CX3CR1 Expression Is Upregulated After Ischemia and Hypoxia, Leading to Neuronal Apoptosis

**FIGURE 3:**
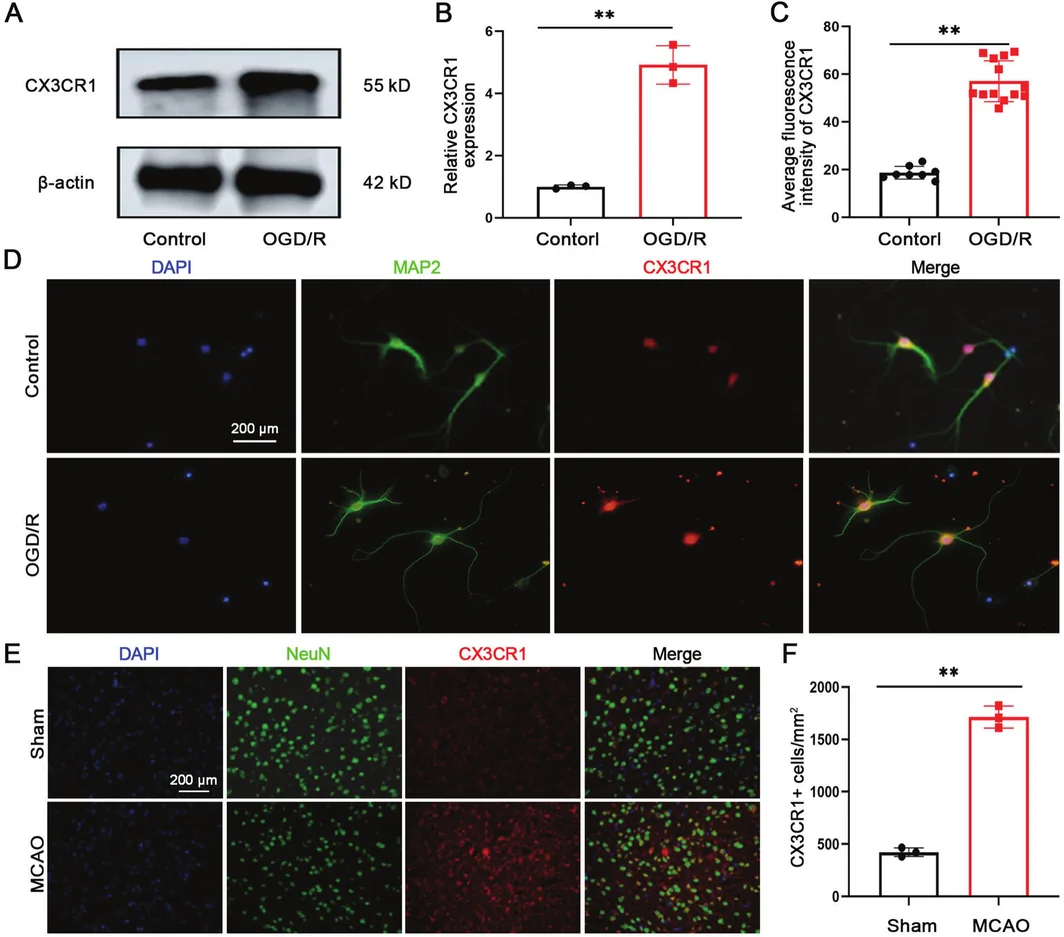
Upregulation of CX3CR1 expression in neurons after ischemia and hypoxia. (A,B) Western blotting analysis of primary neurons showed that the expression of CX3CR1 in primary neurons was upregulated after OGD for 30 min and reoxygenation for 24 h(**p < 0.01, *n* = 3). (C,D) Immunofluorescence analysis of primary neurons showed that the expression of CX3CR1 in primary neurons was upregulated after OGD for 30 min and reoxygenation for 24 h(**p < 0.01, *n* = 10). (E,F) Double staining of CX3CR1 and NeuN showed that CX3CR1 is co‐localized with neurons, and ischemia and hypoxia can lead to upregulation of CX3CR1 expression in neurons. Semi‐quantitative analysis showed a significant increase in the number of CX3CR1/NeuN positive cells after MCAO (**p < 0.01, *n* = 3).

Meucci et al. [13]. and our previous research have demonstrated the expression of CX3CR1 in hippocampal neuronal cells. Building upon this, we investigated the impact of ischemia–hypoxia on neuronal CX3CR1 expression and its association with neuronal apoptosis. Utilizing an OGD/R model with primary neurons in vitro, Western blot analysis revealed a significant upregulation of CX3CR1 expression under hypoxic conditions compared to controls (Figure 3A,B). Additionally, CX3CR1 fluorescence staining indicated a marked increase in CX3CR1 fluorescence intensity following hypoxia, with values rising from (18.72 ± 2.67) in the control group to (57.07 ± 8.56) (Figure 3C,D). Immunofluorescence double staining of NeuN and CX3CR1 further demonstrated a substantial increase in the number of NeuN and CX3CR1 double‐positive cells in the infarcted brain tissue of the MCAO group compared to controls, with values escalating from (263 ± 32/mm^2^) to (1259 ± 91/mm^2^) (Figure 3E,F). Collectively, both in vivo and in vitro findings suggest an upregulation of neuronal CX3CR1 expression following ischemia–hypoxia injury.

**FIGURE 4:**
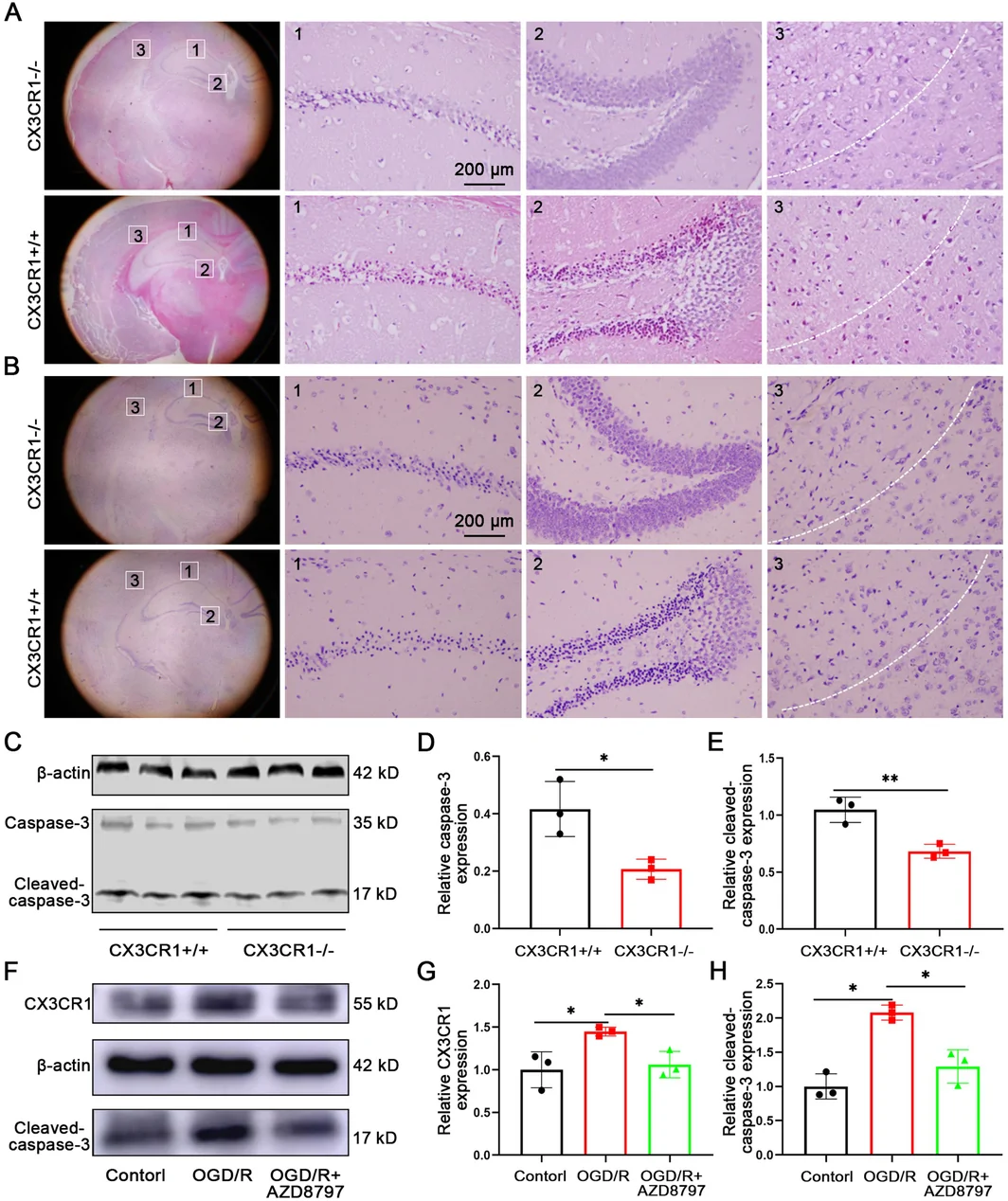
CX3CR1 mediates neuronal apoptosis after ischemia and hypoxia. (A) HE staining of (1) striatum, (2) hippocampus, (3) surrounding area of infarcted brain tissue after CX3CR1^+/+^and CX3CR1^−/−^ MCAO. (B) CX3CR1^+/+^and CX3CR1^−/−^ MCAO, Nissl staining was performed on (1) striatum, (2) hippocampus, and (3) surrounding area of infarcted brain tissue (*n* = 3). (C–E) Western blotting showed that caspase‐3 expression was upregulated after CX3CR1+/+MCAO, while caspase‐3 expression decreased after CX3CR1gene knockout (*p < 0.05, * * *p* < 0.01, *n* = 3). (F–H) Western blotting showed that OGD/R upregulated the expression of CX3CR1 in neurons, while the expression decreased after administration of CX3CR1 inhibitors. The expression trend of Cleaved‐Caspase‐3 in neurons was the same as that of CX3CR1 (*p < 0.05, *n* = 3).

To investigate the impact of CX3CR1 on neuronal apoptosis following ischemia and hypoxia, a middle cerebral artery occlusion (MCAO) model was established using CX3CR1 gene knockout mice and wild‐type (WT) mice. Histological analysis through paraffin section hematoxylin and eosin (HE) staining revealed distinct cellular changes in the infarcted striatum, hippocampus, and surrounding brain tissue regions in both CX3CR1^+/+^ and CX3CR1^−/−^ groups. In the CX3CR1^+/+^ group, cells exhibited characteristics indicative of apoptosis, such as reduced volume, cytoplasmic condensation, presence of apoptotic bodies, enhanced eosinophilic staining, deeply stained nuclei with clumped chromatin, and cell surface “budding.” Conversely, the CX3CR1^−/−^ group displayed fewer apoptotic features, including less cytoplasmic condensation, fewer apoptotic bodies, less noticeable eosinophilic staining, and less deeply stained nuclei. HE staining demonstrated a significant reduction in brain tissue cell apoptosis on the infarcted side following MCAO in CX3CR1 gene knockout mice (Figure 4A). Additionally, Nissl staining was employed to further assess the impact of CX3CR1 on neuronal apoptosis. The Nissl staining results indicated that compared to the CX3CR1^−/−^ group, the CX3CR1^+/+^ group exhibited decreased Nissl body volume and number, along with lighter staining in various regions of the infarcted striatum, hippocampus, and surrounding brain tissue, underscoring the role of CX3CR1 in reducing neuronal apoptosis after MCAO (Figure 4B). Furthermore, Western blot analysis was conducted to evaluate Caspase‐3 levels in the infarcted brain tissue of WT mice and CX3CR1 knockout mice post‐MCAO. The findings revealed a significantly higher expression of apoptotic protein in the infarcted brain tissue of WT mice compared to CX3CR1 knockout mice post‐MCAO (Figure 4C–E).

To exclude the influence of microglia and related cells on neuronal apoptosis, we added CX3CR1 inhibitor AZD8797 during OGD/R reoxygenation of neuronal cells in vitro. Western blotting results showed that OGD/R could upregulate the expression of CX3CR1 and cleaved‐Caspase‐3, which was statistically significant compared with the control group. After the addition of inhibitors, the expression of CX3CR1 was downregulated; the corresponding expression of cleaved‐Caspase‐3 decreased, which was statistically significant compared with the OGD/R group (Figure 4F–H). The above in vitro and in vivo experimental results indicate that ischemia and hypoxia lead to upregulation of neuronal CX3CR1 and are correlated with neuronal apoptosis. Knocking out CX3CR1 can alleviate neuronal apoptosis caused by ischemia and hypoxia.

### 3.3 Mechanism of Scutellarin Directly Alleviating Neuronal Apoptosis Through the HIF‐1α‐CX3CR1 Axis

#### 3.3.1 Scutellarin Downregulates Neuronal CX3CR1 Expression Following Ischemia and Hypoxia Through Modulation of HIF‐1α Expression

**FIGURE 5:**
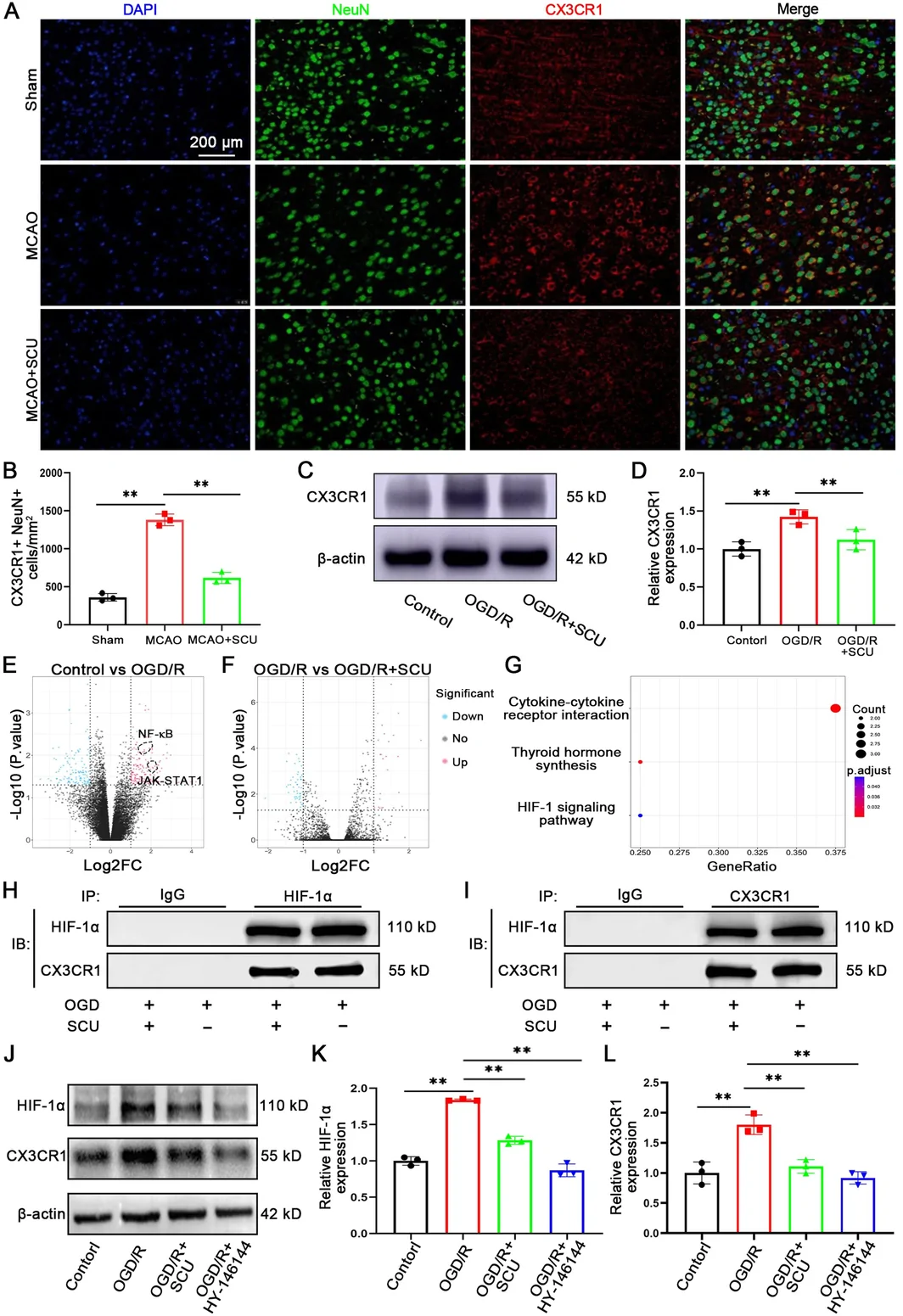
By modulating HIF‐1α expression, scutellarin downregulated CX3CR1 expression in neurons following ischemic and hypoxic conditions. (A) Expression of CX3CR1 in brain tissue of normal C57BL/6 mice (upper panel). Post‐MCAO, neuronal CX3CR1 expression increased in the infarcted cerebral region of C57BL/6 mice (middle panel). Scutellarin treatment reduced the upregulated neuronal CX3CR1 expression (lower panel). (B) Quantitative analysis showed a significant increase in CX3CR1/NeuN‐positive cells post‐MCAO, with scutellarin treatment decreasing this number. (*n* = 3, **p < 0.01). (C,D) Western blotting analysis showed that the expression of CX3CR1 was upregulated in Neuronal cells after OGD/R, while treatment with scutellarin could reduce the expression of CX3CR1. (E,F) The volcano plot displays significantly differentially expressed genes in different groups (control group, model group, SCU group). (G) Enrichment of KEGG pathways for differentially expressed genes. (H,I) Representative CO‐IP images of HIF‐1α and CX3CR1 under the treatment conditions of scutellarin and the ischemia‐hypoxia model. (J–L) Inhibition of HIF‐1α expression can lead to a decrease in CX3CR1 expression (*n* = 3, **p < 0.01).

Increased expression of CX3CR1 in neurons can induce neuronal apoptosis, whereas scutellarin has been shown to mitigate neuronal apoptosis. We investigated whether scutellarin exerts its anti‐apoptotic effects by downregulating CX3CR1 expression in neurons. Paraffin‐embedded mouse brain sections were double immunofluorescence stained for NeuN and CX3CR1. Our findings revealed a reduction in the number of neurons in the infarcted hemisphere compared to the control group. CX3CR1 was colocalized with NeuN, and the number of CX3CR1‐positive neuronal cells significantly increased (1,381 ± 62/mm^2^), a statistically significant difference compared to the control group (334 ± 56/mm^2^). In the scutellarin treatment group, intraperitoneal administration of scutellarin markedly decreased the number of apoptotic neurons and reduced CX3CR1 expression on neurons (615 ± 60/mm^2^), a statistically significant difference compared to the MCAO group (Figure 5A,B). Protein level analysis following in vitro OGD/R demonstrated that OGD/R upregulated CX3CR1 expression in neurons compared to the control group, with a statistically significant difference between the two groups (Figure 5C,D). Scutellarin treatment attenuated the upregulation of CX3CR1 induced by OGD/R, with a statistically significant difference between the two groups.

To delve deeper into the underlying mechanisms, we cultured primary neurons in vitro across three experimental groups: Control, OGD/R, and SCU. Subsequent transcriptome sequencing unveiled 999 differentially expressed genes between the model and control groups, comprising 486 upregulated and 513 downregulated genes. Within the treatment group vs. the model group, 201 differentially expressed genes were identified, with 71 upregulated and 130 downregulated genes. Notably, several of these genes are implicated in the NF‐κB and JAK1‐STAT1 pathways (Figure 5E,F). KEGG pathway analysis highlighted HIF‐1 as a shared upstream regulator (Figure 5G). Previous research has demonstrated that in diabetic retinopathy, scutellarin can mitigate oxidative stress and enhance HIF‐1α degradation [14]. Additionally, studies have shown that HIF‐1α modulates inflammatory cell infiltration and cancer cell migration through CX3CR1 [15]. Consequently, we sought to investigate whether scutellarin could modulate the NF‐κB and JAK1‐STAT1 pathways via the HIF‐1α‐CX3CR1 axis to mitigate neuronal apoptosis following ischemia and hypoxia. Co‐immunoprecipitation (IP) assay confirmed the physical interaction between HIF‐1α and CX3CR1 in Neuronal cells under the treatment conditions of scutellarin and the ischemia‐hypoxia model (Figure 5H,I). The in vitro experiments using the HIF‐1α inhibitor (HY‐146144) also confirmed the regulatory association between HIF‐1α and CX3CR1 across different experimental groups: The control, OGD/R, OGD/*R* + SCU, and OGD/R + HY‐146144 groups. Western blot analysis demonstrated that scutellarin treatment notably decreased the levels of HIF‐1α and CX3CR1, exhibiting a significant difference compared to the OGD/R group. Treatment with the HIF‐1α inhibitor (HY‐146144) significantly reduced HIF‐1α levels, leading to a corresponding decrease in CX3CR1 expression, which was significantly different from the OGD/R group (Figure 5J–L). These combined in vivo and in vitro findings suggest that scutellarin mitigates CX3CR1 expression in neurons following ischemia and hypoxia by modulating HIF‐1α expression.

#### 3.3.2 Scutellarin Inhibits Neuronal Apoptosis by Regulating the NF‐κB Pathway Through HIF‐1α‐CX3CR1

**FIGURE 6:**
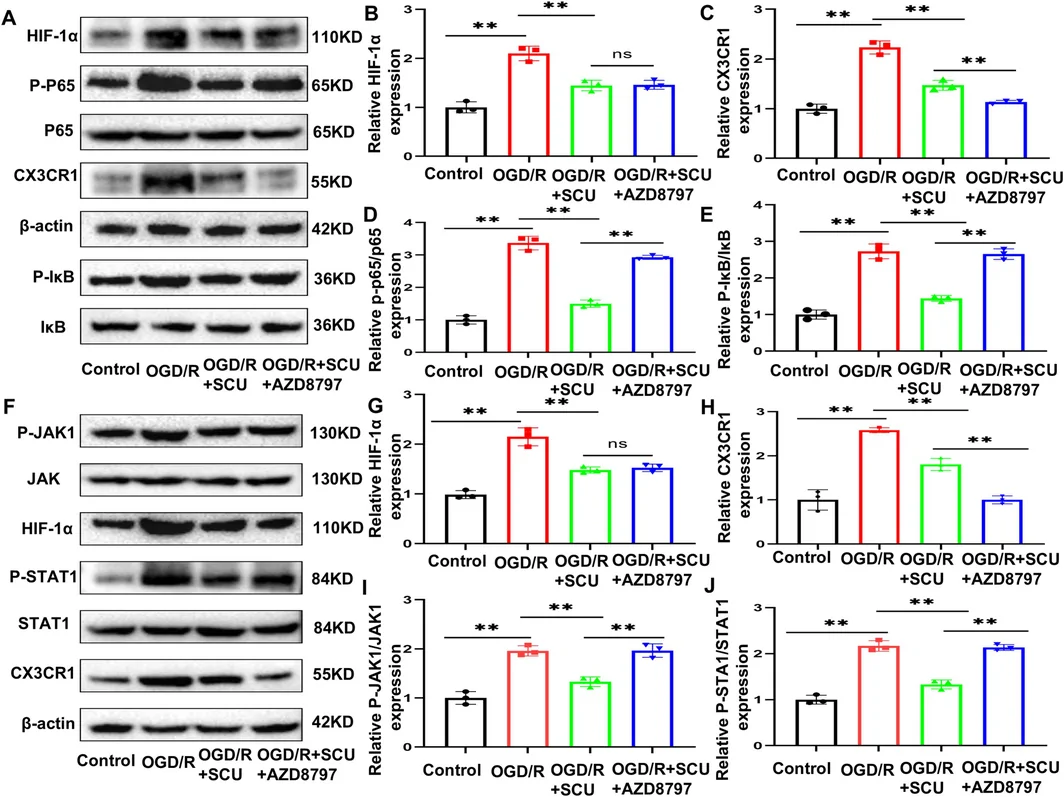
Scutellarin inhibits neuronal apoptosis by regulating the NF‐κB/JAK1‐STAT1 pathway through the HIF‐1α‐CX3CR1 axis. (A–E) Representative Western blot bands showing protein levels of HIF‐1α, CX3CR1, P‐IκB, IκB, P‐P65 and P65 following treatment with scutellarin and CX3CR1 inhibitors. Quantification of HIF‐1α, CX3CR1, P‐IκB, P‐P65 protein levels by Western blotting (*p < 0.05, **p < 0.01, *n* = 3). (F–J) Representative Western blot bands showing protein levels of HIF‐1α, CX3CR1, P‐JAK1, JAK1, P‐STAT1, and STAT1 following treatment with scutellarin and CX3CR1 inhibitors. Quantification of HIF‐1α, CX3CR1, P‐JAK1 and P‐STAT1 protein levels by Western blotting (*p < 0.05, **p < 0.01, *n* = 3).

**FIGURE 7:**
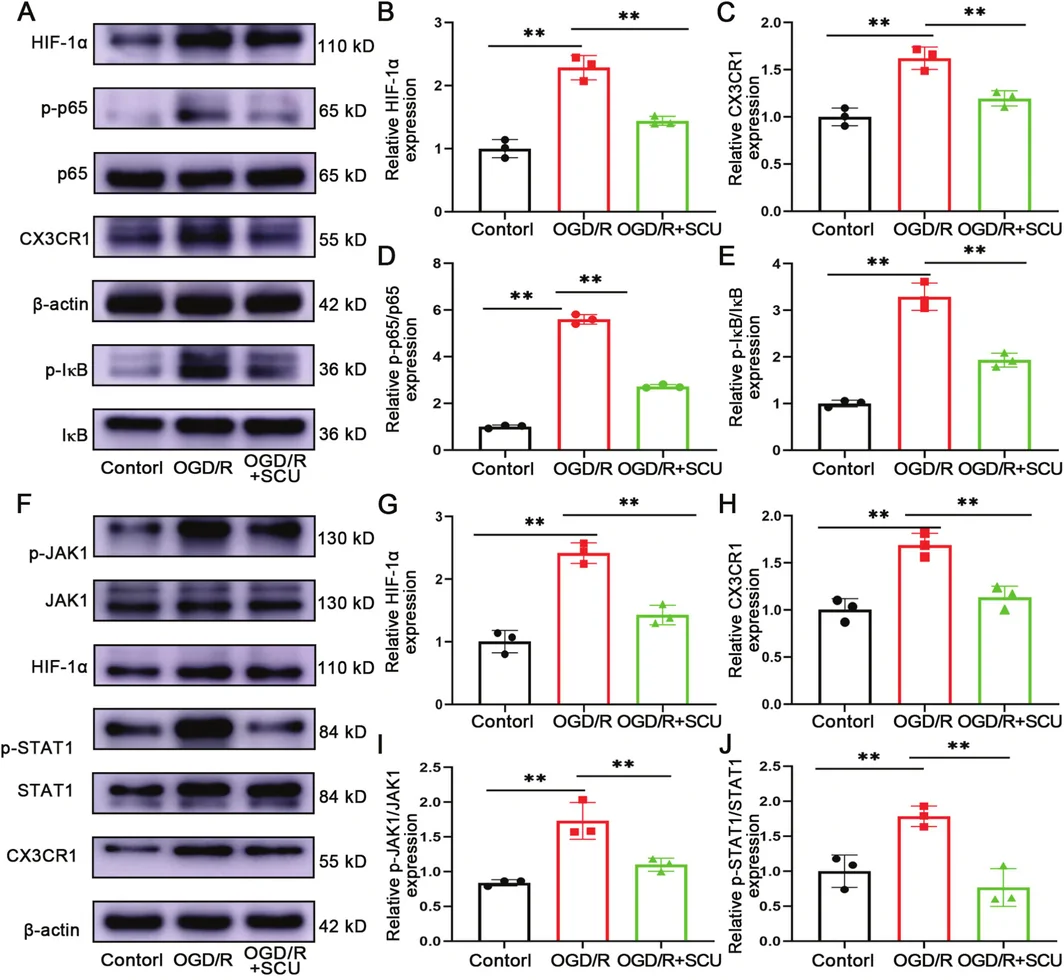
Scutellarin inhibits neuronal apoptosis by regulating the NF‐κB/JAK1‐STAT1 pathway through the HIF‐1α‐CX3CR1 axis. (A–E) Representative Western blotting images showed the expression of HIF‐1α, CX3CR1, P‐IκB, IκB, P‐P65, and P65 proteins. Quantification of HIF‐1α, CX3CR1, P‐IκB, P‐P65 protein levels by Western blotting (*p < 0.05, **p < 0.01, *n* = 3). (F–J) Representative Western blotting images showed the expression of HIF‐1α, CX3CR1, P‐JAK1, JAK1, P‐STAT1, and STAT1 proteins. Quantification of HIF‐1α, CX3CR1, P‐JAK1 and P‐STAT1 protein levels by Western blotting (*p < 0.05, **p < 0.01, *n* = 3).

Multiple experimental findings suggest an association between the NF‐κB pathway and apoptosis. Our previous research has confirmed that CX3CR1 can regulate NF‐κB‐related pathways (Figure S2) [16]. However, limited research has explored whether scutellarin can mitigate neuronal apoptosis by modulating the NF‐κB pathway via the HIF‐1α–CX3CR1 axis. We further investigated the effects of the CX3CR1 inhibitor (AZD8797) in combination with scutellarin. Protein analysis revealed that CX3CR1 blockade could eliminate the downstream effects of the NF‐κB pathway induced by scutellarin, without affecting upstream regulators HIF‐1α and CX3CR1 expression levels (Figure 6A–E). Western blot analysis of pivotal NF‐κB pathway proteins demonstrated that, after scutellarin treatment, there was a significant decrease in the levels of HIF‐1α, CX3CR1, p‐IκB/IκB, and p‐P65/P65 compared to the OGD/R group. These outcomes suggest that scutellarin modulates NF‐κB pathway proteins through the HIF‐1α–CX3CR1 axis, thereby ameliorating neuronal apoptosis following ischemia and hypoxia (Figure 7A–E).

#### 3.3.3 Scutellarin Inhibits Neuronal Apoptosis by Regulating the JAK1‐STAT1 Pathway Through HIF‐1α‐CX3CR1

Recent studies have shown that the JAK1‐STAT1 pathway is implicated in tumor cell proliferation and apoptosis. To explore the potential of scutellarin in regulating neuronal apoptosis via this pathway, we conducted in vitro experiments using neuronal cells treated with varying concentrations of the CX3CR1 inhibitor, AZD8797. Western blot analysis revealed a dose‐dependent decrease in the phosphorylation levels of JAK1 and STAT1 with increasing AZD8797 concentration. Moreover, pre‐treatment with different concentrations of the STAT1 activator HY‐W013523 reversed the inhibitory effects of AZD8797 on JAK1 and STAT1 phosphorylation levels (Figure S3). We further investigated the effects of the CX3CR1 inhibitor (AZD8797) in combination with scutellarin. Protein analysis revealed that CX3CR1 blockade could eliminate the downstream effects of the JAK1‐STAT1 pathway induced by scutellarin, without affecting upstream regulators HIF‐1α and CX3CR1 expression levels (Figure 6F–J). Further Western blot analysis demonstrated that following scutellarin treatment, protein levels of HIF‐1α, CX3CR1, p‐JAK1/JAK, and p‐STAT1/STAT1 decreased significantly compared to the OGD/R group. These findings support the notion that scutellarin modulates proteins associated with the JAK1‐STAT1 pathway through the HIF‐1α–CX3CR1 axis, thereby mitigating neuronal apoptosis post‐ischemia–hypoxia (Figure 7F–J). This study unveils, for the first time in a neuronal ischemia–hypoxia model, the involvement of this pathway, offering a novel theoretical framework for utilizing scutellarin in ischemic stroke treatment and identifying a potential therapeutic target for clinical intervention in ischemic stroke.

## 4 Discussion

Risk factors for ischemic stroke include age, hypertension, diabetes, atrial fibrillation, and hypercholesterolemia. Regardless of the triggering event, ischemic stroke deprives brain cells of oxygen and energy, leading to cellular metabolic disorders at the molecular level. The mechanisms of ischemic brain injury comprise excitotoxicity, oxidative stress, nitrosative stress, inflammation, and apoptosis [17]. Once ischemia and hypoxia occur, cells, especially neurons, cannot maintain normal function. Subsequently, ischemic brain tissue can release inflammatory cytokines, increase production of oxygen free radicals and excitatory neurotransmitters, and disrupt the blood–brain barrier [18]. Inhibiting these pathophysiological processes can alleviate cellular damage. Therefore, exploring new mechanisms of neuronal apoptosis and seeking new neuroprotective agents aimed at improving neurological prognosis have become an important focus, attracting the attention of researchers worldwide.

Previous studies have shown that the protective effect of scutellarin on nerve cells is mainly achieved through molecular mechanisms involving anti‐oxidative stress and inhibition of apoptosis. Regarding its antioxidant effects, scutellarin can increase the activities of superoxide dismutase and glutathione peroxidase and reduce malondialdehyde (MDA) levels in brain tissue, thereby alleviating oxidative stress damage to nerve cells [19]. With respect to inhibiting apoptosis, scutellarin protects nerve cells by modulating apoptosis‐related protein expression, including reducing Caspase‐3 and Caspase‐9 activities, increasing the Bcl‐2/Bax ratio, and decreasing cytochrome c (Cyt‐c) release [20]. These findings indicate that scutellarin inhibits apoptosis through multiple pathways. However, few studies have directly examined the protective effect of scutellarin on neuronal cells. Most of the studies have focused on exerting neuroprotective effects by regulating the polarization states of microglia and astrocytes and reducing the secretion of inflammatory factors [21]. The results of this experiment show that scutellarin can reduce the apoptosis of neurons caused by ischemia and hypoxia, confirming that scutellarin has a direct protective effect on neurons.

CX3CR1 is a member of the G protein‐coupled receptor (GPCR) family. CX3CR1 plays an important role in the central nervous system and is involved in regulating the pathophysiological processes of multiple disease models. In the mild controlled cortical impact injury model, injured wild‐type mice exhibited more severe manifestations in terms of motor deficits, cell death, and neuronal cell loss than the CX3CR1 knockout mice [22]. In the epilepsy model, increased expression of CX3CR1 was detected in samples from patients with temporal lobe epilepsy and experimental animal models. Blockade using an anti‐CX3CR1 antibody reduces the activation of microglia, neuronal death, and the generation of neural stem cells in the hippocampus of adult rats caused by electrical seizures [23]. In summary, CX3CR1 has distinct roles across various disease models. Previous studies on CX3CR1 in ischemic stroke have primarily investigated how reducing the release of inflammatory mediators from microglia exerts neuroprotective effects. Few studies have investigated neuronal expression of CX3CR1 in the ischemia‐hypoxia model. This study is the first to confirm that neuronal expression of CX3CR1 is upregulated after ischemia‐hypoxia and that CX3CR1 knockout reduces neuronal apoptosis.

In ischemic stroke, cells are subjected to hypoxia and energy deficiency, which activate a series of biological response mechanisms. Among them, hypoxia‐inducible factors have attracted extensive research attention. Hypoxia‐Inducible Factor (HIF) refers to a class of protein transcription factors that play a crucial role when cells respond to a hypoxic environment. HIF is a heterodimer composed of two subunits, namely Hypoxia‐Inducible Factor 1α (HIF‐1α) and Hypoxia‐Inducible Factor 1β (HIF‐1β). The expression level of HIF‐1α is mainly regulated by oxygen levels, while HIF‐1β is relatively stable [24]. HIF‐1α can affect the proliferation, apoptosis, and migration of tumor cells, as well as treatment regimens, through many different signaling pathways [25]. The research on HIF‐1α and CX3CR1 mainly focuses on tumors. Some studies have confirmed that hypoxia upregulates the expression of CX3CR1 in pancreatic cancer cells. When the expression of Hypoxia‐Inducible Factor (HIF)‐1α is knocked down in vitro and in vivo, the expression of CX3CR1 is significantly reduced. Chromatin immunoprecipitation assay shows that HIF‐1α binds to the hypoxia response element (HRE; 5′‐A/GCGTG‐3′) of the CX3CR1 promoter under normoxic conditions, and this binding is significantly enhanced under hypoxic conditions. HIF‐1α may regulate cancer cell migration through CX3CR1 [26]. Tissue microarrays of human ovarian cancer cells show elevated expression of CX3CR1 and HIF‐1α, and their levels are even higher in stages II and III of adenocarcinoma [27]. There is no relevant research on whether HIF‐1α can regulate the expression of CX3CR1 in neurons. Our experimental results show that HIF‐1α can regulate the expression of CX3CR1 in neurons under ischemic–hypoxic conditions. Furthermore, scutellarin treatment reduces the expression of CX3CR1 by regulating HIF‐1α.

Another key event in ischemic stroke and neurotoxicity is the activation of nuclear factor kappa B (NF‐κB). NF‐κB is a transcription factor involved in signaling pathways that activate inflammasomes, and the activation of NF‐κB is associated with the degree of brain injury [28, 29]. The four‐vessel occlusion model in rats shows that ischemia–hypoxia can lead to the activation of NF‐κB, which is closely related to neuronal apoptosis in the hippocampus [30]. The MACO model in rats indicates that transient focal cerebral ischemia leads to the activation of NF‐κB in neurons, which promotes neuronal apoptosis [31]. Thus, activation of NF‐κB appears to promote neuronal apoptosis. There are few studies on the correlation between CX3CR1 and NF‐κB. Blocking NF‐κB in the sciatic neuralgia model downregulates CX3CL1–CX3CR1 signaling. Conversely, the CX3CR1 neutralizing antibody also downregulates p‐NF‐κB [32]. There is no relevant research on whether CX3CR1 on neurons can affect neuronal apoptosis through the NF‐κB/p65 pathway. The results of this experiment indicate that scutellarin regulates neuronal apoptosis through the HIF‐1α–CX3CR1–NF‐κB pathway.

Janus kinases (JAKs) are a class of tyrosine kinases that regulate biological processes, including cell growth, development, and immune responses, via phosphorylation. The Janus kinase family comprises JAK1, JAK2, JAK3, and Tyk2. They have distinct functions across different cell types and biological processes [33]. Studies on the relationship between CX3CR1 and JAK/STAT have shown that in human smooth muscle cells, CX3CR1 can affect the progression of human atherosclerosis through the JAK1/STAT1 pathway [34]. CX3CR1 can upregulate the inflammatory response of AR42J cells through the JAK/STAT pathway [35]. There is no relevant research on whether CX3CR1 on neurons can affect their apoptosis through the JAK1/STAT1 pathway. We were the first to confirm this pathway in a neuronal ischemia–hypoxia model, providing a novel theoretical basis for scutellarin's use in treating ischemic stroke and identifying a new target for its clinical management.

The limited understanding of neuronal apoptosis after ischemic stroke hinders preventative strategies due to a lack of imaging techniques and targeted therapies. Future methods may include hypothermia and neuroprotective agents to extend treatment windows and identify the penumbra. Administering neuroprotective agents before hospital admission could halt ischemic progression, providing more treatment options. Management may involve neuroprotective, thrombolytic, and neurorestorative agents to protect tissue and restore blood flow.

## 5 Conclusion

This study reveals that neuronal CX3CR1 expression is elevated following ischemia and hypoxia, and genetic deletion of CX3CR1 confers protection against ischemic stroke. Mechanistically, scutellarin reduces CX3CR1 mRNA levels by modulating HIF‐1α, leading to decreased CX3CR1 protein expression. CX3CR1 is implicated in regulating neuronal apoptosis post‐ischemia through the NF‐κB/P65 and JAK1‐STAT1 pathways. Key findings of this investigation, building upon prior research, include: (1) Direct neuroprotective effects of scutellarin on neuronal cells. (2) Upregulation of CX3CR1 in neurons in an ischemia‐hypoxia model, correlating with neuronal apoptosis. (3) Scutellarin's regulation of NF‐κB/P65 and JAK1‐STAT1 pathways in neurons post‐ischemia and hypoxia via the HIF‐1α‐CX3CR1 axis, influencing apoptosis. These results offer a foundation for the clinical utilization of scutellarin and present potential therapeutic targets for ischemic stroke management.

## Author Contributions

Conceived the study and experimental design:Zhiwei Tang, Jingchun Pei. Performed experiments: Jingchun Pei, Bin Hu, Cheng Wan. Analyzed data: Zhonghui Wen, Fuqing Zhang, Shaoxiang Li, Lei Wang, Zhigao Li. Wrote the manuscript: Jingchun Pei, with significant input from all co‐authors. Zhiwei Tang made revisions to the initial draft. The project is funded by Jingchun Pei and Zhiwei Tang. All authors read and approved the manuscript.

## Funding

This work was supported by the Yunnan Provincial Department of Education Science Research Fund Project (2025J0189), the Doctoral Research Launch Fund of the First Affiliated Hospital of Kunming Medical University (2024BS001), the National Natural Science Foundation of China (82360246), the Yunnan Applied Basic Research Projects (202301AY070001‐008), the 535 Talent Project of First Affiliated Hospital of Kunming Medical University (2022535D04), and the Yunnan Clinical Medical Center for Neurocardiac Diseases (zx2019030501).

## Ethics Statement

Animal experiments performed in the present study were approved by Kunming Medical University (approval no. Kmmu202220920) and were conducted in accordance with international guidelines for animal studies and the guidelines of the Committee on Animal. All animal husbandry and experiments were strictly conducted in accordance with the management and use regulations of laboratory animals.

## Conflicts of Interest

The authors declare no conflicts of interest.

## Supporting information


**Table S1:** Longa scoring criteria.
**Table S2:** Primer sequences.
**Figure S1:** Purity identification of primary neurons (400X).
**Figure S2:** Expression of JAK1‐STAT1 pathway in nerve cells. Western blotting (A–F) was used to detect the expression of p‐JAK1/JAK and p‐SATAT1/STAT1 in neuronal cells. (*p < 0.05, **p < 0.01, *n* = 3).
**Figure S3:** Expression of the JAK1‐STAT1 pathway in nerve cells. Western blotting (A–F) was used to detect the expression of p‐JAK1/JAK and p‐SATAT1/STAT1 in neuronal cells. (*p < 0.05, **p < 0.01, *n* = 3).

## Data Availability

The data that support the findings of this study are available from the corresponding author upon reasonable request.
