# Supplementary material for: Scutellarin Alleviates Neuronal Apoptosis After Ischemia and Hypoxia via the HIF‐1α–CX3CR1 Axis
Source: CNS Neurosci Ther. 2026 Aug 19;32(8):e71098. doi: 10.1002/cns.71098 (PMC13487807; doi:10.1002/cns.71098)
Supplement: Supplementary file 1 [file CNS-32-e71098-s001.docx]

Table S1: Longa scoring criteria

| **Score** | **Neurological symptoms** |
| --- | --- |
| 0 points | No neurological deficits |
| 1 points | Left forelimb extension disorder |
| 2 points | Go left in a circle |
| 3 points | Tilt to the left |
| 4 points | Can't walk on their own, unconscious |

Table S2: Primer sequences

| Gene ID | primer sequences |
| --- | --- |
| CX3CR1 F | CTGGACGCCTTACAACATCG |
| CX3CR1R | TCTGAACTTTTCCCCAGCGA |
| β-ACTIN F | ACCGCAAATGCTTCTAAACC |
| β-ACTIN R | CCAATCTCGTCTTGTTTTATGC |

Figure S1 : Purity identification of primary neurons (400X)

Figure S2: Expression of JAK1-STAT1 pathway in nerve cells. Western blotting (A-F) was used to detect the expression of p-JAK1/JAK and p-SATAT1/STAT1 in neuronal cells. （*p <0.05,**p < 0.01,n=3）.

Figure S3: Expression of JAK1-STAT1 pathway in nerve cells. Western blotting (A-F) was used to detect the expression of p-JAK1/JAK and p-SATAT1/STAT1 in neuronal cells. （*p <0.05,**p < 0.01,n=3）.
